# A Successful Approach to Diagnosing Shiga-like Toxin-Producing *Escherichia coli*-Induced Colitis

**DOI:** 10.3390/diagnostics14080801

**Published:** 2024-04-11

**Authors:** Violeta Melinte, Adelina M. Radu, Cristina M. Văcăroiu, Miriana I. Cismaru, Anca M. Oprescu Macovei, Daniela E. Mihăilă, Valeriu Gheorghiță

**Affiliations:** 1 Faculty of Medicine, Carol Davila University of Medicine and Pharmacy, 020021 Bucharest, Romania; maria-adelina.cosma@drd.umfcd.ro (A.M.R.); valeriu.gheorghita@umfcd.ro (V.G.); 2 Agrippa Ionescu Clinical Emergency Hospital, 011356 Bucharest, Romaniam_e_d_iv@yahoo.com (D.E.M.)

**Keywords:** STEC, bloody diarrhea, ischemic colitis, *E. coli*

## Abstract

Shiga-like toxin-producing Escherichia coli (STEC) is a well-known cause of foodborne acute diarrheic diseases, especially in children and the elderly. The potentially fatal complications associated with toxin production range from bloody diarrhea and ischemic colitis to kidney failure, hemolytic–uremic syndrome (HUS), and colon perforation. Here, we describe a case and literature review of STEC-induced colitis, highlighting the clinical features and the necessary tools for the best diagnostic approach and management. Facing challenging differential diagnosis, ranging from ischemic colitis and inflammatory bowel disease to infectious processes due to a pathogenic or opportunistic agent, we conducted a step-by-step exploration. Following bacteriological investigation, imagistic screening, and colonoscopy, we ruled out some of the initial suppositions and reached a final diagnosis, while also considering the pathological results. Although antibiotics are not indicated in this pathology, our patient did receive antibiotics, given the risk of translocation and colon perforation, without any associated complications such as HUS or peritonitis. Detailed and rigorous investigations conducted by a multi-specialty team are required for prompt medical support. Coping with the symptoms and refraining from further complications are the mainstem aims of treatment.

## 1. Introduction

*Escherichia coli* strains are commonly found in the human and animal gastro-intestinal (GI) tract [1]. There are several groups that are pathogenic to humans: enterotoxigenic *E. coli* (ETEC), enteropathogenic *E. coli* (EPEC), enterohemorrhagic *E. coli* (EHEC), ente-roinvasive *E. coli* (EIEC), and enteroaggregative *E. coli* (EAEC). Some strains of EHEC can produce Shiga-like toxins (STEC), responsible for hemolytic–uremic syndrome (HUS) and ischemic colitis [2]. There are two primary serotypes of enterohemorrhagic *E. coli*: O157:H7 and non-O157:H7; both serotypes generate toxins resembling those of *Shigella* [3], but the most severe cases are linked to serotype O157:H7 [4]. STEC-induced infections are often self-limited or mild, and they do not require antibiotics or surgical intervention. Pathogenic strains can also colonize asymptomatic humans, posing a risk to their contacts—both children and adults [5].

This paper aims to highlight the relevance of diagnostic tools in approaching digestive pathology, as well as the importance of a multi-disciplinary team that can successfully manage this disorder.

We reviewed eight cases of STEC-induced colitis in adults reported in the literature in the last 12 years, published on PubMed, in gastroenterology journals, or in medical case reports in scientific publications. The keywords used were ischemic colitis, STEC, and *E. coli*.

We report a case of colitis in a patient with no other comorbidities who recovered after a 10-day course of antibiotics without developing HUS or requiring surgical intervention.

## 2. Case Presentation

A 64-year-old woman was admitted to our clinic with bloody diarrhea, intensive abdominal pain, and loss of appetite with a two-day-earlier onset. She had no relevant medical history. The patient reported no smoking, alcohol consumption, or substance abuse. She denied having any autoimmune illness or inflammatory bowel disease in her family. She used to intermittently visit her two-year-old niece.

Two months and two weeks earlier, respectively, the patient’s daughter-in-law and niece had experienced digestive symptoms like flatulence, abdominal pain, and diarrhea. They both had confirmed enterohemorrhagic *E. coli* non-O157:H7 strains detected in their stool cultures, and both recovered after antibiotic therapy without being hospitalized. 

On physical examination, the patient had a normal weight, was afebrile, and had a pulse of 90 bpm and blood pressure of 115/74 mmHg. An abdominal exam showed reduced bowel movements and stabbing pain, given a score of 8 out of 10 in severity, without any guarding. She declared frequent bloody loose stools, up to 10–15 times a day.

The laboratory findings revealed leukocytosis (21,100/mm^3^, with the normal range values being 4000–10,000/mm^3^), mild abnormal electrolytic values, mildly increased inflammatory markers (CRP 1.4 mg/dL, with the normal range values being 0–0.5 mg/dL) and fibrinogen (486 mg/dL with the normal range values being 200–400 mg/dL), and no other remarkable abnormalities (Table 1).

**Figure 1:**
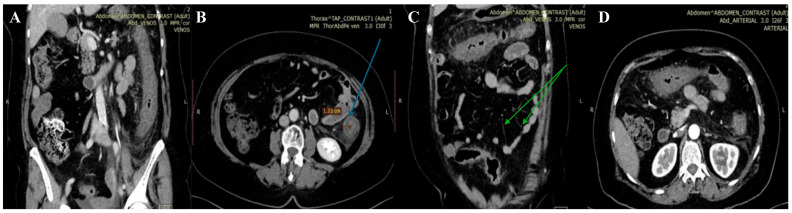
First abdominal CT. Spastic large intestine, with thickened circumferential mucosa (blue arrow), up to 1.33 cm (**B**), and an incomplete “comb” sign (green arrow) (**C**). Coronal (**A**,**C**) and axial planes (**B**,**D**) respectively.

**Figure 2:**
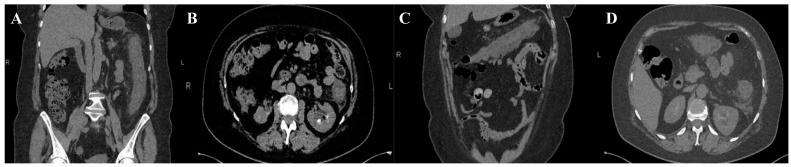
Second abdominal CT. Extensive and continuous lesions, up to hepatic flexure and down to sigmoid with pericolic fat stranding. Coronal (**A**,**C**) and axial planes (**B**,**D**) respectively.

Microbiological investigations ruled out *Clostridium difficile*, *Shigella*, *Salmonella*, and parasitic involvement, and a PCR-based multiplex pathogen GI test detected *Shiga*-like toxin-producing *Escherichia coli* (STEC) stx1/stx2 genes. The STEC serotype and subtype could not be determined. The abdominal CT revealed a spastic transverse and descendent large intestine with a thickened circumferential mucosa, suggestive of an inflammatory and infectious process, which progressed over the following days (Figure 1 and Figure 2).

Despite supportive treatment consisting of rehydration and symptomatic drugs, her condition worsened the next day, both clinically and biologically (Table 1).

**Figure 3:**
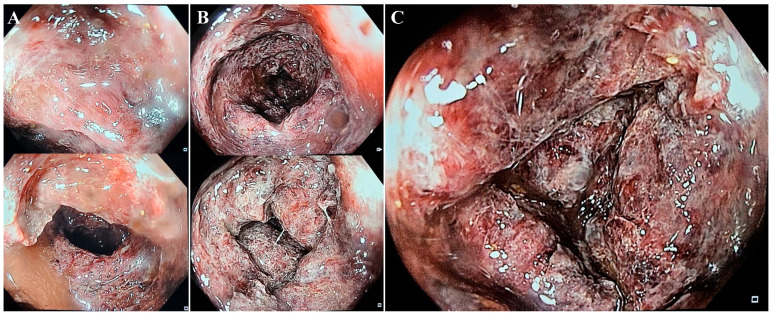
Sigmoid colon—quasi-normal aspect with erythematous lesions and hemorrhagic debris (**A**). Descending colon—sections of erythematosus mucosa, with multiple ulcerations (**B**). Descending colon at 60 cm from anal canal—deep ulcerated lesions and friable, hemorrhagic mucosa (**C**).

On the second day, the patient underwent a colonoscopy that revealed a less-affected sigmoid colon, with deep ulcerations, dusky areas, and necrotic lesions in the ascending colon, as well as friable mucosa located 40–60 cm from the anal canal (Figure 3), an atypical picture for inflammatory bowel disease. Because of the friability of the damaged mucosa, the gastroenterology specialist could not advance any further through the transverse colon with the colonoscope.

**Figure 4:**
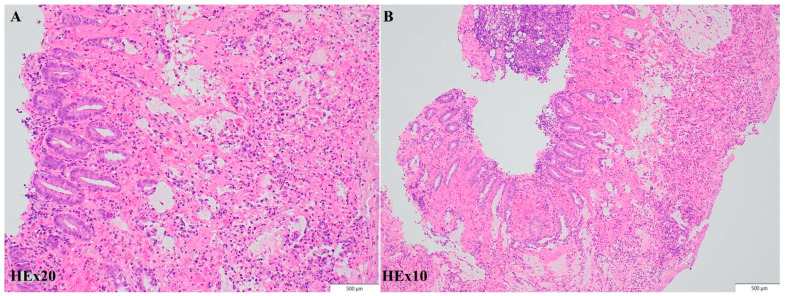
Histopathologic findings. Moderate to severe injury of the colonic mucosa, ranging from acute inflammation associated with focal superficial hemorrhagic lesions to lamina propria necrosis and cryptic damage with fibrinous exudate and vascular congestion, compatible with acute colitis. Hematoxylin-eosin; original magnification ×20 (**A**), ×10 (**B**) respectively.

The histological report described focal superficial necrotic lesions in the colonic mucosa, covered by fibrin and hemorrhagic debris, and deep hemorrhagic lesions with cryptic destruction consistent with ischemic colitis due to infectious agents (Figure 4).

On the third day, the patient underwent a second abdominal CT scan, which showed extensive colonic lesions up to the hepatic flexure and down to the sigmoid and rectum (Figure 2), preserving the mesenteric artery with contrast medium. This picture was compatible with the micro-ischemic lesions found in infectious ischemic colitis [6,7].

It was a significant challenge to determine whether the patient was experiencing infectious or ischemic colitis at the beginning. Her clinical condition seemed more like ischemic colitis, but the biological parameters (LDH, CK, D-dimer) did not sustain this supposition (Table 1). Conversely, CT scans revealed inflammatory changes without any large vessel occlusion. The colonoscope images revealed multiple necrotic lesions and inflammatory and hemorrhagic exudate, similar to those found in ischemic colitis. Tissue biopsies were finally able to determine micro-ischemic and necrotic intestinal changes, probably due to an infectious agent. The histopathological findings did not correlate with inflammatory bowel disease, nor with hemorrhagic colitis due to CMV reactivation. Other infectious agents can cause hemorrhagic or ischemic colitis, like *Campylobacter*, *Salmonella*, *Clostridium difficile*, and *Shigella*, but none of these were found using PCR-based testing or stool culture sampling. The final diagnosis was ischemic colitis due to Shiga-like toxin-producing *Escherichia coli*.

Given the unfavorable progress in the first three days and the high risk for bacterial translocation, the patient was given a 10-day course of tigecycline and fluconazole. At the same time, she was transferred from the infectious disease clinic to the surgery clinic, since it was possible that she could have required a total colectomy.

The patient continued receiving all parenteral nourishment and bowel rest. After maintaining supportive therapy and antibiotics, she recovered over the following days. She was discharged with an improved clinical condition, having normal intestinal transit with no biological inflammation. She was advised to undergo a repeat CT scan and/or colonoscopy after three months.

## 3. Discussion

Shiga-like toxin-producing Escherichia coli (STEC) is a varied group of *E. coli* that may produce over 200 different serotypes of Shiga toxins. Significant outbreaks of human sickness, ranging in severity from simple diarrhea to severe consequences of hemorrhagic colitis and hemolytic uremic syndrome (HUS), are caused primarily by the foodborne bacterium O157 STEC. Several non-O157 STEC serotypes have become significant foodborne pathogens globally and are responsible for outbreaks and occasional cases of foodborne illness with severity comparable to that of O157 STEC. An increased risk of STEC infection also arises from direct contact with infected animals, such as those seen in petting farms and zoos, particularly in young children [8].

Shiga toxin 1 (Stx1) and/or Stx2 production is the defining characteristic and a primary virulence factor in all STEC strains. These two enzymes are further classified phylogenetically into three subtypes of Stx1 (a, b, and d) and seven subtypes of Stx2 (a, b, c, d, e, f, and g). STEC that produces Stx2 is more frequently linked to severe human disorders such as HUS and hemorrhagic/ischemic colitis, particularly the three Stx2 subtypes Stx2a, Stx2c, and Stx2d [9].

It is a considerable challenge to try to differentiate between ischemic colitis per se and that induced by infectious agents. Laboratory tests, microbiological studies, and cultures for parasitic or bacterial involvement are the first tools used [10]. Polymerase chain reaction (PCR)-based molecular methods are new-generation tests that can be helpful and time-sparing, yet having their limits. In our case, the only bacteriological results were PCR-based. The final diagnosis was not culture-proven and lacked the ability to determine the type or serotype, but one can speculate on intra-familial transmission. The two members of the patient’s family were successfully treated for enterocolitis due to non-O157:H7 EHEC with amoxicillin–clavulanate and cefuroxime, respectively, according to their very distinct antibiograms. They most probably did not share the same *E. coli* strain. It is very unlikely, but not impossible, that our patient became infected with one of these strains. Considering that there was no food incriminated in this case, our patient might have been an asymptomatic STEC carrier until this episode. 

Tomographic features very suggestive of bacterial colitis are (1) continuous distribution, (2) an empty colon, (3) the absence of fat stranding, (4) the absence of a “comb” sign, and (5) the absence of enlarged lymph nodes [2]. According to these criteria, our patient had extensive and continuous distribution, collapsed intestines, and the absence of enlarged lymph nodes. However, contrarily, she had pericolic fat stranding and an incomplete “comb” sign, which made the final diagnosis more difficult to determine.

Rectoscopy or colonoscopy and tissue biopsies complete the investigational plan for achieving a positive diagnosis. In our case, although the entire colon was affected, the endoscope could reveal no further than 40–60 cm from the anal canal because of the risk of iatrogenic colon perforation. Histological biopsies from the colon or rectum may show similar patterns to those in ischemic colitis, such as atrophic crypts, coagulative necrosis of the mucosa, hyalinized lamina propria, and fibrin thrombi [6].

The management of STEC-induced colitis does not require antibiotics but fluid replenishment and supportive care. Some studies conducted on animal models highlight the benefits of alternative treatment such as monoclonal antibodies aimed at Shiga toxins, toxin receptor analogs, or vaccines [11]. Antibiotics may increase the risk of hemolytic–uremic syndrome by releasing more toxins while killing bacteria, especially in children [3]. Despite this consideration, the risk of the translocation of endogenic bacteria through the affected mucosa was too high to be ignored in the presented case. While treatment for acute ischemic colitis typically results in resolution, individuals who require surgery still have significant rates of mortality, up to 44.9%, when emergency colectomy is performed [12]. Facing a potential lethal complication such colon perforation, peritonitis, or abdominal sepsis, we chose an antibiotic with good intestinal penetration, which could cover a large bacterial spectrum, including STEC. At a 4-month follow-up, our patient was in good condition, and the colonoscopy revealed restitutio ad integrum. At this time, the stool culture showed no STEC strain.

There are few published case reports on ischemic colitis due to Shiga-like toxin-producing *E. coli* in adults (Table 2) [7,13,14,15,16,17,18,19]. Among these reports, the most commonly found clinical features were abdominal pain and bloody diarrhea, with or without fever. Laboratory findings revealed unspecific changes such as mild to intense leukocytosis or slight inflammation. Microbiological assessments provided STEC-positive stool cultures in four out of eight cases [7,14,17,18], and a CT scan was performed in six of the cases.

Seven out of eight cases had a colonoscopy performed (Table 3) with tissue biopsy, with the remaining case exhibiting macro-/micro-histological findings due to exploratory laparotomy [16]. In a single patient, the ileum mucosa was exclusively affected [14], and another case had extremely severe necrosis up to the transverse colon, making further examination impossible, similar to our case [13]. In the remainder of the cases, the intestinal mucosa damage was arbitrarily scattered all along the colon, sparing the rectum in three patients, possibly affecting the right side to a greater degree.

Two patients with colon necrosis [17,18] and one with fecal peritonitis [16] underwent surgical interventions with favorable outcomes. All the patients received antibiotics for at least one day, including quinolones, cephalosporins, penicillin, and metronidazole, which are known for their risk of triggering HUS, but only two patients of extreme age developed HUS [18,19].

Hemolytic–uremic syndrome, a known clinical condition associated with progressive kidney failure due to microangiopathic changes including hemolytic anemia and thrombocytopenia [20], was especially found in children with acute diarrheic disease due to STEC, according to some studies conducted in Romania between 2010 and 2016 [21,22]. The studies revealed a high STEC prevalence (6.4%) in symptomatic hospitalized children aged up to 30 months old. Almost one out of four developed HUS. In 2021, there were 6534 cases of STEC colitis reported by 30 EU/EAA countries. Out of these, there were 365 HUS cases reported, with the majority being in the youngest age group of 0–4 years (64%) [8].

## 4. Conclusions

STEC-induced colitis should be considered in patients with bloody diarrhea and abdominal pain, either with or without fever. Laboratory testing should consist of hematological evaluation, extensive culture of stools on MacConkey agar medium, and PCR-based multiplex if available. CT scans can rule out obstructive or/and oncologic lesions and ischemic changes to large arteries and could indicate acute or chronic inflammatory patterns. Colonoscopy should be performed in all severe cases whose cause has not been confirmed. Even though the treatment mainly involves supportive measures, antibiotics can also be cautiously given when anticipating bacterial translocation through an affected intestinal wall. Surgical measures cannot be disregarded when patients’ clinical and biological scenarios, CT scans, or colonoscopy evidence indicate imminent complications such as colonic perforation.

## Figures and Tables

**Table 1:** Laboratory values during hospitalization.

	Day 1	Day 3	Day 6	Day 8	Day 10
WBC (10^3^/mm^3^)	21.1	18	12.5	9.5	8.3
Neutrophils (%)	77.9	81.4	76	71	60
Platelets (10^3^/mm^3^)	331	238	238	255	326
Hemoglobin (g/dL)	14.9	12	11.8	11.4	11.8
Fibrinogen (mg/dL)	483	641	469	363	
CRP (mg/dL)	1.4	98.9	69.82	30	
BUN (mg/dL)	25		37		
Creatinine (mg/dL)	0.5	0.5	0.6	0.68	0.5
Na (mmol/L)	133	132			140
K (mmol/L)	3.48	3.56		4.06	4.67
BT (mg/dL)	1.0			0.17	
BD (mg/dL)	0.2			0.06	
BI (mg/dL)	0.8				
AST (U/L)	18			41	19
ALT (U/L)	23	15		28	17
GGT (U/L)	27			59	
ALP (U/L)	74				
Amylase (U/L)	68		27	23	
Lipase (U/L)	13				
Albumin (g/dL)			2.67		3.34
Glucose (mg/dL)	146		73	65	
LDH (U/L)		192			
CK (U/L)		22			
D-DIMER (ng/mL)		1665			
PCT (ng/mL)		<0.5			

BUN—blood urea nitrogen; BT—bilirubin total; BD—bilirubin direct; BI—bilirubin indirect; ALP—alkaline phosphatase; PCT—procalcitonin.

**Table 2:** Literature review.

Reference	Cases (Sex, Age)	Clinical Findings	Biological Changes	Microbiological Tests	Imagistic Investigations	Histological Findings	Management	HUS	Other Complications
Kravitz 2022 [16]	F, 48	Pain, fever, vomiting, non-bloody diarrhea	Leucocytosis, low sodium and potassium levels	Negative stool culture; antibodies to *E. coli* O157 LPS with positive dynamics	Ileus and free subdiaphragmatic air on radiography	Bowel wall rupture, acute hemorrhagic colitis with ischemic features	Imipenem–cilastatin; piperacillin–tazobactam; fluconazole; surgical intervention	No	Colon perforation
Kendrick 2007 [17]	M, 59	Pain, fever, bloody diarrhea	Mild leukocytosis	*E. coli* O157:H7 isolated in stool culture	Wall thickening through the entire colon at CT scan	Inflammatory pseudo-membranes, mucosal ischemia, and ulceration	Metronidazole, surgical intervention	No	Colon necrosis
Tominaga 2014 [18]	M, 81	Pain, febrile, bloody diarrhea,	Inflammatory syndrome, thrombocytopenia	*E. coli* O157 isolated in stool culture; positive verotoxin	Thickening of the entire colon wall and ascites at CT scan	Hemorrhagic necrosis into mucosa with subjacent oedema	Cefotiam, levofloxacin, surgical intervention	Yes	Colon necrosis, septic shock
Radhakrishnan 2019 [19]	M, 17	Pain, fever, bloody diarrhea	Mild leukocytosis,	Positive STEC antibodies	Wall thickening in the ascending colon at CT scan	Hemorrhagic lesions, inflammatory exudate, and atrophic crypts	Cefuroxime, metronidazole, eculizumab	Yes	Thrombocytopenia, partial-complex seizures
Tanquilut 2019 [13]	F, 32	Pain, afebrile, non-bloody diarrhea	Intense leukocytosis, low sodium	GI panel stool positive for STEC, negative stool culture	ND	Superficial mucosal necrosis, hemorrhages into lamina propria	Ciprofloxacin, metronidazole, piperacillin–tazobactam,	No	No
Caldis 2021 [14]	F, 59	Pain, afebrile, non-bloody emesis, dark stool	Moderate leukocytosis	*E coli* O111 isolated in stool culture	Severe colitis, without evidence of large vessel occlusion on CT angiogram	Ischemic colitis in appearance	Ceftriaxone, metronidazole	No	No
Cocca 2022 [15]	M, 44	Asthenia, fever, melena	Mild inflammation (CRP)	GI panel stool positive for STEC O157	Thickened wall of terminal ileum at angio-CT	Inflamed and ulcerated mucosa	Azithromycin	No	No
Al-Smadi 2023 [7]	M, 21	Pain, afebrile, bloody diarrhea	Mild leukocytosis	Stool positive for STEC	Normal CT scan of large intestine	Erosion and necrosis of mucosa, crypt atrophy	Ceftriaxone, metronidazole	No	No

**Table 3:** Endoscopic changes.

No	Reference	Cases (Sex, Age)	Ileum	Ascending Colon	Descending Colon	Rectum
1.	Kravitz 2022 [16]	F, 48	ND	ND	ND	ND
2.	Kendrick 2007 [17]	M, 59	−	++	++	++
3.	Tominaga 2014 [18]	M, 81	−	++	+	−
4.	Radhakrishnan 2019 [19]	M, 17	−	+	+	+
5.	Tanquilut 2019 [13]	F, 32	ND	ND	+++	+
6.	Caldis 2021 [14]	F, 59	−	++	+	+
7.	Cocca 2022 [15]	M, 44	+++	−	−	−
8.	Al−Smadi 2023 [7]	M, 21	−	+	++	−

Abbreviations: F—female; M–male; ND—no data; absence of intestine macroscopic changings (−) or mild (+)/ moderate (++)/ severely affected (+++).

## Data Availability

The authors declare that the data for this research are available from the corresponding authors upon reasonable request.

## References

[1] Beutin L., Fach P. (2014). Detection of Shiga Toxin-Producing Escherichia coli from Nonhuman Sources and Strain Typing. Microbiol. Spectr..

[2] Plastaras L., Vuitton L., Badet N., Koch S., Di Martino V., Delabrousse E. (2015). Acute colitis: Differential diagnosis using multidetector CT. Clin. Radiol..

[3] Tarr P.I., Gordon C.A., Chandler W.L. (2005). Shiga-toxin-producing Escherichia coli and haemolytic uraemic syndrome. Lancet.

[4] Kaper J.B., O’Brien A.D. (2014). Overview and Historical Perspectives. Microbiol. Spectr..

[5] Fernandez-Brando R.J., Sacerdoti F., Amaral M.M., Bernal A.M., Da Rocha M., Belardo M., Palermo M.S., Ibarra C.A. (2023). Detection of plasma anti-lipopolysaccharide (LPS) antibodies against enterohemorrhagic Escherichia coli (EHEC) in asymptomatic kindergarten teachers from Buenos Aires province. Rev. Argent. Microbiol..

[6] Azer S.A., Tuma F. (2023). Infectious Colitis. StatPearls.

[7] Al-Smadi D.M., Shahwan M.Y., Madi M.Y. (2023). Breaking Down the Gut: A Case of Severe Toxin-Mediated Colitis. Cureus.

[8] European Centre for Disease Prevention and Control (2022). STEC Infection. ECDC.

[9] Zhang H., Yamamoto E., Murphy J., Carrillo C., Locas A. (2021). Shiga Toxin-Producing Escherichia coli (STEC) and STEC-Associated Virulence Genes in Raw Ground Pork in Canada. J. Food Prot..

[10] Jessurun J. (2017). The Differential Diagnosis of Acute Colitis: Clues to a Specific Diagnosis. Surg. Pathol. Clin..

[11] Mühlen S., Dersch P. (2020). Treatment Strategies for Infections With Shiga Toxin-Producing *Escherichia coli*. Front. Cell. Infect. Microbiol..

[12] Ryoo S.B., Oh H.K., Ha H.K., Moon S.H., Choe E.K., Park K.J. (2014). The outcomes and prognostic factors of surgical treatment for ischemic colitis: What can we do for a better outcome?. Hepatogastroenterology.

[13] Tanquilut C.D., Jung C.W., Nelson A.W., Lau S.K. (2019). Infection due to Shiga toxin-producing enterohemorrhagic *Escherichia coli* (EHEC) presenting as ischemic colitis. IDCases.

[14] Caldis M., Austin K., Slukvin I. (2021). S1915 A Sporadic Case of Shiga Toxin-Producing Enterohemorrhagic Escherichia Coli O111 Infection Manifesting as Ischemic Colitis. Am. J. Gastroenterol..

[15] Cocca S., Campigotto M., Simonini M.S., Gozzi C., Grande G., Mangiafico S., Bertani H., Conigliaro R. (2022). Enterohemorrhagic shiga- like toxin producer Escherichia Coli Ileitis: The first capsule endoscopy report in adults. J. Clin. Images Med. Case Rep..

[16] Kravitz G.R., Smith K., Wagstrom L. (2022). Colonic Necrosis and Perforation Secondary to *Escherichia coli* O157:H7 Gastroenteritis in an Adult Patient without Hemolytic Uremic Syndrome. Clin. Infect. Dis..

[17] Kendrick J.B., Risbano M., Groshong S.D., Frankel S.K. (2007). A rare presentation of ischemic pseudomembranous colitis due to Escherichia coli O157:H7. Clin. Infect. Dis..

[18] Tominaga T., Oikawa M., Takeshita H., Kunizaki M., Tou K., Abo T., Hidaka S., Nanashima A., Sawai T., Nagayasu T. (2014). Successful Colectomy for Hemorrhagic Colitis with Hemolytic Uremic Syndrome and Acute Encephalopathy due to *Escherichia coli* O157 Infection. Case Rep. Gastroenterol..

[19] Radhakrishnan S.T., Ruban A., Uthayakumar A.K., Cohen P., Levy J., Teare J. (2019). Haemolytic uraemic syndrome—A rare case report of bloody diarrhoea in adults. BMC Gastroenterol..

[20] Michael M., Bagga A., Sartain S.E., Smith R.J.H. (2022). Haemolytic uraemic syndrome. Lancet.

[21] Falup-Pecurariu O., Lixandru R.I., Cojocaru E., Csutak K., Monescu V., Muhsen K., Falup-Pecurariu C., Cohen D. (2019). Shiga toxin producing *Escherichia coli*-associated diarrhea and hemolytic uremic syndrome in young children in Romania. Gut Pathog..

[22] Peron E., Zaharia A., Zota L.C., Severi E., Mårdh O., Usein C., Bălgrădean M., Espinosa L., Jansa J., Scavia G. (2016). Early findings in outbreak of haemolytic uraemic syndrome among young children caused by Shiga toxin-producing *Escherichia coli*, Romania, January to February 2016. Eurosurveillance.

